# Genome-wide association and linkage analyses localize a progressive retinal atrophy locus in Persian cats

**DOI:** 10.1007/s00335-014-9517-z

**Published:** 2014-04-29

**Authors:** Hasan Alhaddad, Barbara Gandolfi, Robert A. Grahn, Hyung-Chul Rah, Carlyn B. Peterson, David J. Maggs, Kathryn L. Good, Niels C. Pedersen, Leslie A. Lyons

**Affiliations:** 1 Department of Population Health and Reproduction, School of Veterinary Medicine, University of California - Davis, Davis, CA 95616 USA; 2 College of Science, Kuwait University, 13060 Safat, Kuwait; 3 Department of Veterinary Medicine and Surgery, College of Veterinary Medicine, University of Missouri-Columbia, E109 Vet Med Building, 1600 E. Rollins St., Columbia, MO 65211 USA; 4 College of Medicine, Chungbuk National University, Chongju, Chungbuk Province South Korea; 5 Department of Surgical and Radiological Sciences, School of Veterinary Medicine, University of California - Davis, Davis, CA 95616 USA; 6 Department of Medicine and Epidemiology, School of Veterinary Medicine, University of California - Davis, Davis, CA 95616 USA

## Abstract

Hereditary eye diseases of animals serve as excellent models of human ocular disorders and assist in the development of gene and drug therapies for inherited forms of blindness. Several primary hereditary eye conditions affecting various ocular tissues and having different rates of progression have been documented in domestic cats. Gene therapy for canine retinopathies has been successful, thus the cat could be a gene therapy candidate for other forms of retinal degenerations. The current study investigates a hereditary, autosomal recessive, retinal degeneration specific to Persian cats. A multi-generational pedigree segregating for this progressive retinal atrophy was genotyped using a 63 K SNP array and analyzed via genome-wide linkage and association methods. A multi-point parametric linkage analysis localized the blindness phenotype to a ~1.75 Mb region with significant LOD scores (*Z* ≈ 14, *θ* = 0.00) on cat chromosome E1. Genome-wide TDT, sib-TDT, and case–control analyses also consistently supported significant association within the same region on chromosome E1, which is homologous to human chromosome 17. Using haplotype analysis, a ~1.3 Mb region was identified as highly associated for progressive retinal atrophy in Persian cats. Several candidate genes within the region are reasonable candidates as a potential causative gene and should be considered for molecular analyses.

**Electronic supplementary material:**

The online version of this article (doi:10.1007/s00335-014-9517-z) contains supplementary material, which is available to authorized users.

## Introduction

The eye is a highly complex organ comprised of several highly specialized cells. The development, structure, and function of the eye involves the interaction of thousands of genes. Genetic mutations in genes involving the eye are likely to be detrimental to the fitness of cats, especially random-bred cats. As of 2012, 232 genetic eye conditions have been mapped to a genomic location in humans and 192 **loci** associated with vision abnormalities have been identified (https://sph.uth.edu/retnet/), including some genes with multiple mutations.

Progressive retinal atrophy (PRA) is a generalized term for a range of hereditary conditions that cause retinal dystrophy or degeneration in animals. The conditions generally involve a progressive degeneration of retinal photoreceptors with variations in the age of onset and rate of progression (Petersen-Jones 1998). Cats are positioned to be excellent models for genetic retinal disorders due to the similarity between the structure of the cat’s eye and that of the human (reviewed in Narfstrom et al. 2013). Narfstrom et al. (2013) reviewed ten vision disorders in cats, two were characterized as retinal degenerative diseases. Two additional PRAs have been identified in Persian (Rah et al. 2005) and Bengal cats (Unpublished observation, Lyons 2014).

The first retinal degeneration characterized in cats was an autosomal recessive form with late-onset seen in Abyssinian cats. A single base-pair intronic polymorphism in *CEP290* causes a 4 bp mRNA insertion resulting in a premature stop codon in the transcript (Menotti-Raymond et al. 2007). This feline disease is similar to the human disease, retinitis pigmentosa (RP). The mutation causing the second retinal degeneration disease reported in Abyssinian cats was identified in *CRX* (Menotti-Raymond et al. 2010). A single base-pair deletion was identified in exon 4 of the gene causing a frame shift and a premature stop codon. This rod-cone dysplasia of Abyssinian cats largely resembles Leber Congenital Amaurosis or severe cone-rod dystrophy in humans.

Previous investigations clinically, histologically, and genetically described an early-onset form of PRA in Persian cats (Rah et al. 2005). Clinically, Persian PRA is characterized by reduced pupillary light reflexes as early as 2–3 weeks of age. Rapid disease progression is observed resulting in a near complete loss of photoreceptors by 16–17 weeks of age. Genetically, the disease is autosomally inherited in a recessive mode. Ongoing clinical studies and genetic analyses, including the current study, have been completed using the extension of the pedigree segregating for Persian PRA from the original study.

The present study was designed to localize the causative variant of PRA in Persian cats and to examine the power of the feline pedigree and the 63 K **SNP** array using different techniques of genetic mapping, including parametric linkage analyses and genome-wide association analysis. Using genome-wide genotypic data from the family segregating for the disease and various genome-wide analyses, the causative variant was localized to a ~1.3 Mb region of cat chromosome E1. Inspection of the region revealed at least twenty-two eye-related genes that are viable candidate genes for genetic analysis.

## Materials and methods

### Remapping array single nucleotide polymorphisms (SNPs) to version 6.2 cat genome assembly

The cat DNA array was generated using SNPs that had been localized and spaced on an early assembly of the cat genome (Mullikin et al. 2010). For this study, the SNPs were relocalized on the newest build of the cat genome to support the association studies. The 2011 cat genome reference (ICGSC Felis_catus 6.2/felCat5), including repeat elements, was obtained from the UCSC browser (http://genome.ucsc.edu/). The file (feltCat5.2bit) was used as the source file for downstream analysis. The input sequences of the SNPs on the illumina Infinium Feline 63 K iSelect DNA array (illumina Inc., San Diego, CA) were obtained from the design sequences provided by illumina in the “opa file.” The sequences flanked each SNP with a length of ~100 bp, for a total of ~200 bp. The *BLAT* algorithm was performed on a local host server. A BLAT search was conducted on each SNP’s sequence with all parameter values set as defaults (95 % or greater sequence similarity of 40 bases or longer sequence size).

The following criteria were used to reassign the SNP positions based on the new version of the cat genome assembly and the BLAT results; (i) only search outputs with the highest match score without gaps were considered, (ii) flanking SNP sequences were assigned to chromosomes based on the highest match score, (iii) the chromosomal location was adjusted and the SNP location was defined by averaging the start and end positions of the query sequence in the target sequences, and (iv) SNP sequences that lacked a match in the new genome assembly were assigned to chromosome “unknown” and SNP sequences that had a match score in random segments of unassembled regions were also assigned to chromosome “unknown.”

### Pedigree and clinical description

Animal husbandry and housing were conducted according to University of California—Davis Institutional Animal Care and Use Committee regulations (IACUC protocol #16691). A multi-generation pedigree was previously developed to determine the mode of inheritance and define the clinical presentation of the Persian PRA (Rah et al. 2005). This original pedigree was expanded by continual backcross breedings to maintain the PRA and also with outcrossing to various breeds to increase genetic diversity. The pedigree under analysis was composed of 202 individuals (Fig. S1). Affected individuals were identified by complete neuro-ophthalmic examinations performed as previously described (Rah et al. 2005) by board-certified veterinary ophthalmologists at the Ophthalmology Service of the University of California—Davis, William H. Pritchard Veterinary Medical Teaching Hospital.

### Cat samples

Individuals (*n* = 126) were selected from the pedigree (*n* = 202) for SNP genotyping (Fig. S1). The individuals selected comprised 17 founders, four cats with a single known parent, and 105 offspring with both parents known. DNA was collected, isolated, and prepared as previously described (Gandolfi et al. 2012). Genomic DNA samples (~600 ng each) were submitted to Neogene, Inc. (Lincoln, NE, USA) for SNP genotyping on the illumina Infinium Feline 63 K iSelect DNA array (illumina Inc., San Diego, CA).

### Quality control for the genotypic data

Quality control analyses for the genotypic data of samples and SNP markers were conducted using *PLINK* (Purcell et al. 2007). For cat samples, the following quality controls were applied: (i) individuals with genotyping success rates <80 % were removed from downstream analyses using function (–*mind 0.2*), (ii) sex inconsistencies between genotypes and reported sex were examined using the function (–*check*-*sex*) and individual cats with sex inconsistencies were removed, and (iii) individuals with >5 % Mendelian errors in their corresponding trios were removed. For SNP markers, the following quality controls were applied: (i) SNPs with genotyping rate <90 % were removed using function (–*geno 0.1*), (ii) SNPs on the X-chromosome that exhibited haploid heterozygous genotypes were removed, (iii) SNPs with >5 % Mendelian errors in the pedigree (81 trios) were removed and remaining SNPs with Mendelian errors (<5 %) were set to 0 (no genotype) when present in specific individuals, and (iv) a minor allele frequency of 0.05 was chosen to prune the remaining of SNP data using the function (–*maf 0.05*).

### Genome-wide linkage analysis

Multi-point parametric linkage analyses were performed using the *MERLIN 1.1.2* pedigree analysis package (Abecasis et al. 2002). The dataset was divided into multiple families to reduce the complexity of the pedigree (Table 1). Families with two parents present in the dataset were included (number of families = 25). Linkage analyses were performed on the 18 autosomal chromosomes individually to reduce computation time and the output was later combined for representation.

**Table 1:** Family structure of cats analyzed for the PRA locus

Family no.	Affection		Number of offspring		No. discordant	
	Sire	Dam	Affected	Unaffected	Trios	Sib-pairs
1	∓	∓	1	1	1	1
2^a^	−/−	∓	6	4	6	24
3^a^	−/−	∓	2	5	2	10
4^a^	−/−	∓	3	0	3	0
5^a^	−/−	∓	1	2	1	2
6^a^	−/−	∓	6	3	6	18
7^a^	∓	−/−	2	3	2	6
8^a^	∓	−/−	2	6	2	12
9^a^	∓	−/−	1	1	1	1
10^a^	∓	−/−	2	2	2	4
11^a^	−/−	∓	3	0	3	0
12^a^	∓	−/−	1	1	1	1
13	∓	∓	2	1	2	2
14^a^	−/−	+/+	0	3	0	0
15^a^	+/+	−/−	0	3	0	0
16^a^	−/−	+/+	0	3	0	0
17^a^	−/−	+/+	0	4	0	0
18	+/+	+/+	0	1	0	0
19	∓	+/+	0	1	0	0
20^a^	∓	−/−	0	1	0	0
21^a^	+/+	−/−	0	1	0	0
22^a^	∓	−/−	1	0	1	0
23	+/+	+/+	0	1	0	0
24^a^	+/+	−/−	0	1	0	0
25^a^	+/+	−/−	0	1	0	0
26	–	−/−	2	2	0	4
27	–	–	0	2	0	0
28	–	∓	1	0	0	0
29	–	∓	0	2	0	0
30	–	–	0	2	0	0
Total	Unaff. singleton parent = 12 Aff. Singleton parent = 1		36	57	33	85

^a^Phenotypically discordant parent pairs (*n* = 20). Founders may have been used in more than one family but highlighted once. The first 25 families have two known parents

Parametric linkage analysis was performed using the function (–*model*). A recessive model was used for the disease, assuming a disease allele frequency of 1 % and complete penetrance. The LOD score was estimated for each SNP and the maximum heterogeneity LOD score was selected for presentation. Only informative families, breedings of an affected to a carrier cat or breedings of two carriers, with multiple phenotyped individuals, were included in the linkage analyses.

### Genome-wide association analyses (TDT, sibTDT, and case–control association)

Genome-wide analyses were performed in PLINK (Purcell et al. 2007). The transmission disequilibrium test (TDT) (Spielman et al. 1993) was performed using 33 phenotypically discordant trios using the function (–*tdt*) (Table 1). A TDT among sib-pairs (sibTDT) (Spielman and Ewens 1998) was performed on 85 phenotypically discordant sib-pairs in the pedigree using the function (–*dfam*) (Table 1). The sib-TDT analysis was conducted without including the founders in frequency calculation (–*nonfounders*).

A case–control association analysis was first conducted on the Persian cats in the study population (*n* = 106), with 37 cases and 69 controls, assuming that all individuals were unrelated (–*assoc*). The genomic inflation for this initial analysis was obtained using the function (–*adjust*). To correct the genomic inflation and account for possible sample substructuring in the study population, the samples were analyzed using multi-dimensional scaling (–*genome*). Control individuals that lacked apparent clustering (*n* = 20), generally F1 Persian outcrossed cats, were removed from the dataset, and the case–control association was reanalyzed on 86 samples, 37 cases, and 49 controls. Genome-wide significance for each of the analyses (TDT, sib-TDT, case–control) was determined by performing phenotype permutations (*n* = 100,000) over the SNP dataset (–*mperm 100,000*).

### Haplotype analysis and characterization

The genotypic data surrounding the significant associations and the linkage region were extracted and visually inspected (60 SNPs, ~3.6 Mb, from SNP chrA2.13244196 on Chr E1 position 374 to SNP chrUn5.8587754 position 3624560). The region was examined to define haplotype(s) block(s) unique to the cases compared to controls. The SNP genotypes within the single haplotype identified in the cases (26 SNPs) were compared across the dataset.

## Results

### Remapping array SNPs to 6.2 cat genome assembly

Physical locations of the SNPs in the 63 K illumina SNP array were originally defined using the 2008 cat genome assembly (NHGRI/GTB V17e/felCat4), which contained many gaps and unassembled and unassigned contigs (Mullikin et al. 2010). Many SNPs, based on the 2008 assembly, were assigned to unknown chromosomes (*n* = 6893) and many were incorrectly assigned to chromosomes (*n* = 510). To correct for SNP position assignments and to enable proper analyses using the SNPs on the array, the array’s SNPs were reassigned to new locations based on the 2011 cat genome assembly (ICGSG Felis_catus 6.2/felCat5). The majority of SNPs within each chromosome remained on the same chromosome, but 1997 SNPs were relocated to other chromosomes or the unknown category (Table S1).

Approximately 86 % of the SNPs remained on the same chromosomes but with an updated location (Table S1). Chromosome “unknown” SNPs (*n* = 6,709) were successfully assigned to chromosomes in the 2011 genome reference, which constitutes ~10 % of the SNPs on the array. The reassigned, previously “unknown” SNPs, constituted as few as 1.75 % of the final X-chromosome SNPs and as many as 22 % of E1 chromosome SNPs. Lastly, 510 SNPs, previously assigned to chromosomes were reassigned to chromosome “unknown” due to the lack of actual known positions on chromosomes. Table S1 shows a detailed summary of the number of SNPs on each chromosome and Table S2 contains the updated map file.

### Pedigree structure and quality control

A subset of the pedigree (*n* = 126) was selected for genotyping and analyzed via genome-wide analyses. The SNP genotype data were inspected for quality control criteria. Thirteen individuals were removed due to a genotyping success rate <80 % and a single individual was removed because of sex inconsistencies between genotype and reported gender. Five individuals exhibited Mendelian errors >5 % and were removed from the dataset. Therefore, the final dataset was composed of 106 individuals; 37 cases and 69 controls. The final dataset is presented in Fig. S1, where cases are marked by red symbols and controls are marked by blue symbols. Individuals with multiple matings are presented more than once in the pedigree for ease of visualization. The sex ratio was 1:1 with 53 males and 53 females. Fifteen individual founders were in the final study population dataset, where twelve were unaffected parents, one was an affected parent, and two were unrelated singletons. Ninety-one non-founders were in 28 nuclear families (25 nuclear families with two parents, 3 families of only offspring). The final study population was also composed of 33 affected trios, 20 were phenotypically discordant trios. Eighty-five phenotypically discordant sib-pairs were available (Table 1).

The cat DNA array consists of 62,897 markers distributed over 18 autosomal chromosomes, the X-chromosome, and chromosome unknown. SNPs exhibiting genotyping rate <90 % (*n* = 1,647) were removed and 532 X-chromosome SNPs were removed due to detection of haploid heterozygous errors. These SNPs were likely to be in the pseudoautosomal region of the X-chromosome. SNPs showing >5 % Mendelian errors (*n* = 317) across all trios also were removed, as were 12,494 SNPs that exhibited minor allele frequency <0.05. After quality control, 47,907 SNPs were used in downstream analyses where the genotyping rate in the remaining samples was >99 %.

### Genome-wide analyses

#### Parametric linkage

**Fig. 1:**
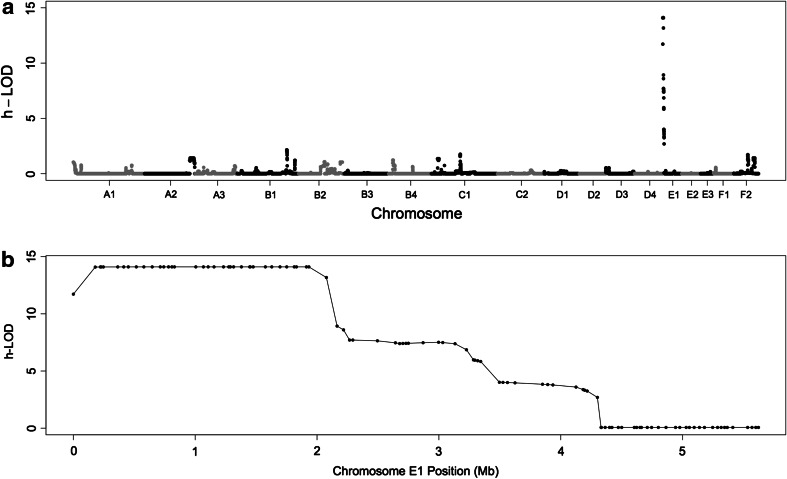
Parametric linkage analysis of Persian cats’ progressive retinal atrophy. **a** Genome-wide LOD scores and **b** a magnified view of the first 5 Mb of chromosome E1

A parametric linkage analysis assuming a recessive model suggested a linkage on chromosome E1 (Fig. 1a). The 40 SNPs with the highest LOD scores are shown in Table S3. The 35 highest SNPs located on chromosome E1 spanned a region of ~1.75 Mb, and exhibited a high LOD score of ~14 with complete linkage. The first 5 Mb of chromosome E1 was markedly linked to the disease (Fig. 1b).

#### Transmission disequilibrium test (TDT)

TDT was performed on 33 phenotypically discordant trios. A strong association was suggested for chromosome E1. Permutation analysis confirmed the association (Figure S2). Ten SNPs with significant *P*_raw_ values are shown in Table 2; all were located on chromosome E1 spanning of ~1.5 Mb region. The SNP with the most significant *P*_raw_ value was chrUn5.6839723, located at position 1,831,172 of chromosome E1 (*P*_raw_ = 4.32E^−8^, *P*_genome_ = 1.0E^−5^).

**Table 2:** Ten most associated markers obtained by genome-wide analyses of Persian PRA

No.	Chr.	SNP ID	Position	TDT		sib-TDT		Case–control	
				*P*_raw_	*P*_genome_	*P*_raw_	*P*_genome_	*P*_raw_	*P*_genome_
1	E1	chrUn5.6839723	1831172	4.32E^−08^	1.00E^−05^	1.00E^−05^	0.1632	7.64E^−13^	1.00E^−05^
2	E1	chrUn5.6133983	1106562	1.21E^−07^	0.00013	0.00013	0.4071	1.52E^−10^	1.00E^−05^
3	E1	chrUn5.6766609	1751066	5.73E^−07^	0.00282	0.00282	0.5451	2.97E^−09^	5.00E^−05^
4	E1	chrUn5.6481762	1452354	7.74E^−06^	0.06134	0.06134	0.9057	2.70E^−07^	0.00226
5	E1	chrUn5.6503134	1476010	2.21E^−05^	0.1905	0.1905	0.9644	1.15E^−06^	0.00841
6	E1	chrUn5.5846986	808916	3.74E^−05^	0.3135	0.3135	0.9487	4.65E^−06^	0.03121
7	E1	chrUn5.6185290	1154788	3.74E^−05^	0.3135	0.3135	0.9487	8.04E^−06^	0.05255
8	E1	chrUn5.6912692	1912858	3.74E^−05^	0.3135	0.3135	0.9793	–	–
9	E1	chrUn5.6942249	1932982	3.74E^−05^	0.3135	0.3135	0.9793	5.95E^−06^	0.03955
10	E1	chrUn5.7293975	2293660	3.74E^−05^	0.3135	0.3135	0.9487	8.76E^−07^	0.00658
11	F1	chrUn5.7948667	2997440	–	–	–	–	4.45E^−06^	0.02986

#### Sibling transmission disequilibrium test (sibTDT)

**Fig. 2:**
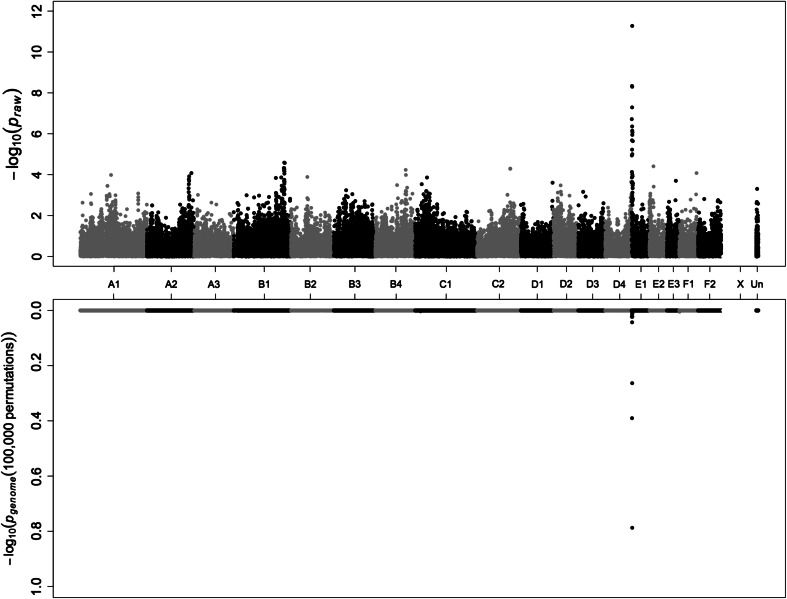
Genome-wide sib-TDT analysis of the Persian PRA. *Upper plot* represents the *P*_raw_ values of the analysis, whereas the *lower plot* represents the genome-wide significant *P*_genome_ values after 100,000 permutations. X-chromosome markers were removed in sib-TDT analysis. Significant association is localized to cat chromosome E1

Sib-TDT analysis was performed on 85 phenotypically discordant sib-pairs. A significant association between the PRA and the SNPs was suggested on chromosome E1 and the location of association with the disease was also significant after permutation (Fig. 2). The ten most significant SNPs are shown in Table 2; all located on chromosome E1. Only three SNPs had a significant *P* value (<0.05) after permutation: chrUn5.6839723, chrUn5.6133983, and chrUn5.6766609 with *P*_genome_ values of 1.0E^−5^, 0.00013, and 0.0028, respectively.

#### Case–control association analysis

Considering all individuals of the study population (*n* = 106) as unrelated, a case–control association analysis was performed (Fig. S3a). The most significantly associated SNP, chrUn5.6839723, was located on chromosome E1 at position 1,831,172. Analysis of all individuals exhibited a genomic inflation (*λ*) of 3.18. To account for the genomic inflation due to population substructure, the individuals were analyzed via multi-dimensional scaling. Twenty unaffected individuals appeared genetically distant from the majority of the individuals (Fig. S4a). These off-clustered unaffected individuals were removed (Fig. S4b), and the case–control association analysis was repeated (Fig. S3b). The most highly associated SNPs are shown in Table 2. The genomic inflation of this modified dataset was measured to be ~1.3. Further reduction of genomic inflation was not possible due to the relatedness of individuals in the pedigree. The average $\hat{P}$ value as a measure of relatedness of all individuals was ~0.18 and, upon removal of the 20 genetically distant samples, the average $\hat{P}$ value was ~0.24.

### Haplotype analysis

The region of highly associated SNPs, which overlapped with the linkage analysis outcome, was investigated for haplotype structure among affected individuals compared to unaffected ones. Visual inspection of the region showed a single haplotype in affected individuals that extended for approximately 1.3 Mb—chrUn5.5751502 (position 713552) to chrUn5.7087155 (position 2076816) (Fig. S5). Three control individuals were homozygous and shared the haplotype of affected individuals but each proved to be a mislabeling in the cat records and blindness was confirmed by the current owners.

### Candidate genes

**Fig. 3:**
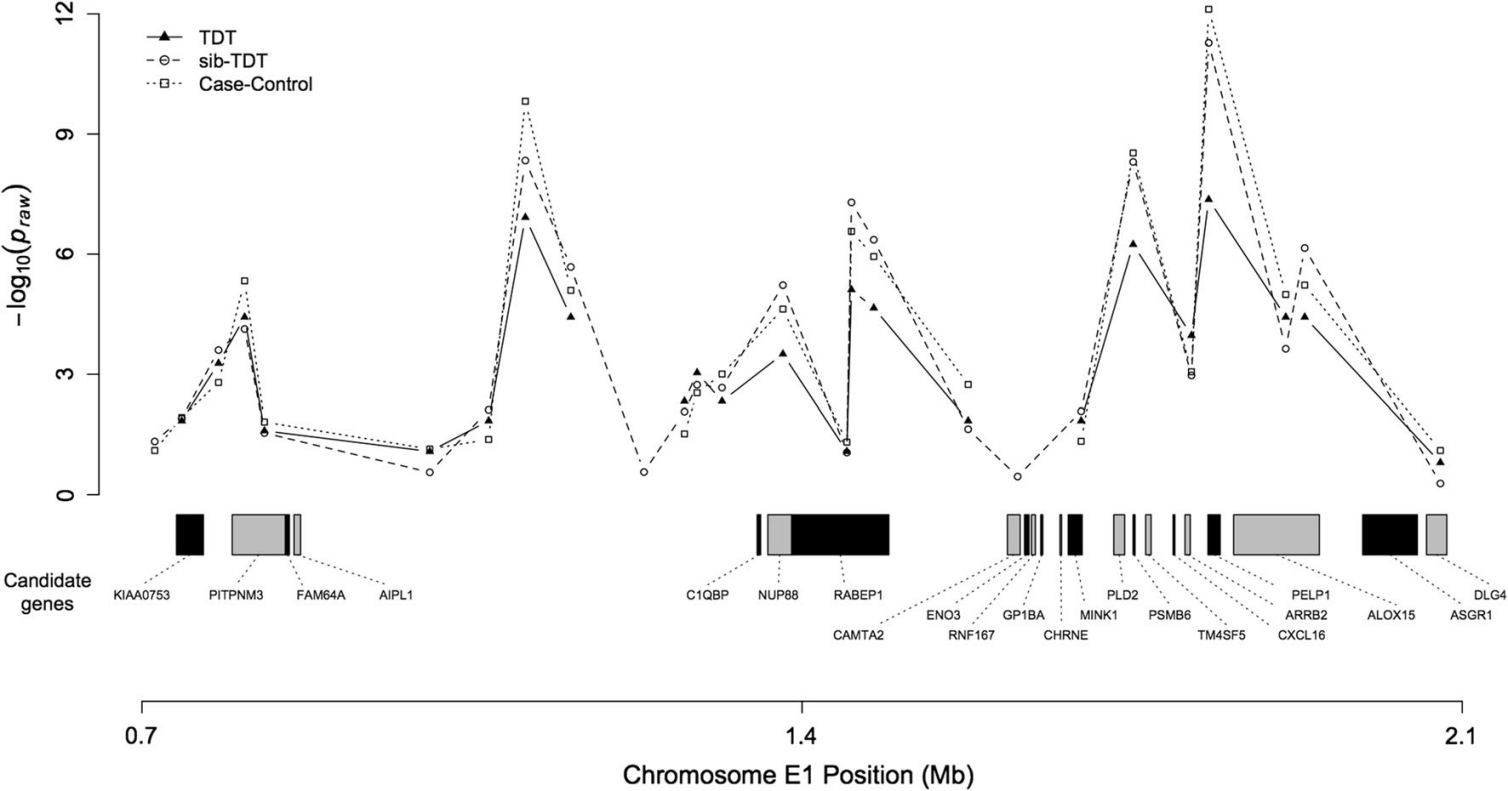
Overview of PPRA region on chromosome E1. The* graph* shows the *P*_raw_ values of the TDT, sib-TDT, and case–control association analyses. Twenty-two candidates genes involved in the retina development or function are presented according to their location

The 1.3 Mb haplotype of affected individuals harbors 42 coding genes (Table S4). Twenty-two of the genes are either associated with the eye or exhibit high expression in the retina. Three candidate genes cause blindness in humans and constitute likely candidates for investigation. *ARRB2* is the closest to the SNP with the highest linkage and association and two high ranking candidate genes (*AIPL1, PIPTNM*) are located more distantly (Fig. 3).

## Discussion

Animal models of human hereditary diseases of the eye are well suited for functional and therapeutic studies. The domestic cat constitutes an ideal model for human eye diseases due to the high similarity in structure and function. Ten ocular disorders have been characterized in cats where analogous human conditions are reported (reviewed in Narfstrom et al. 2013). In particular, two mutations involve degeneration of retinal photoreceptors. Both mutations have been identified via linkage analyses in pedigrees of Abyssinian cats (Menotti-Raymond et al. 2007, 2010).

**Fig. 4:**
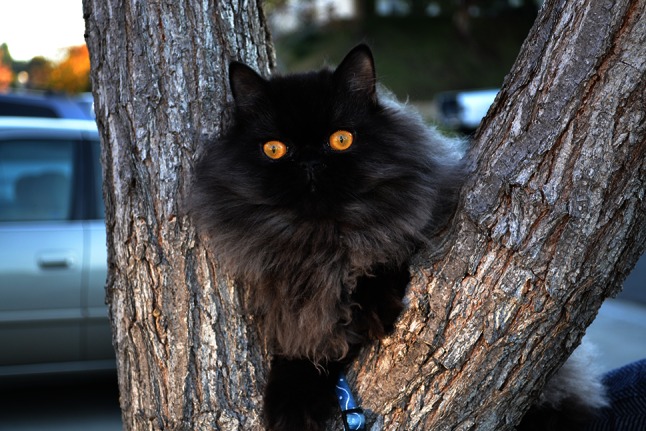
Chocolate Persian cat with progressive retinal atrophy. Persian cats are known for their long hair coat and the brachycephalic structure. The Persian breed is one of the oldest in the cat fancy and one of the most popular worldwide. Persian cats are often used as outcrosses to modify the head structures in newly developing breeds

In this study, a PRA of Persian cats was investigated via a variety of genome-wide analyses. The clinical description of the disease indicates an early-onset condition with rapidly progressive photoreceptor degeneration leading to complete loss of vision by 16–17 weeks of age (Rah et al. 2005). The disease is autosomally inherited in a recessive mode, with complete penetrance. Persian cats are one of the oldest and well-known cat breeds worldwide, as well as one of the most popular (Fig. 4). This brachycephalic breed is often used to modify the facial structures of more modern breeds that are under development (Filler et al. 2012). Thus, not only will Persian cats benefit from a genetic test to eradicate the condition from the breeding population, but also new breeds using Persian stock for modification can avoid inadvertent introduction of this deleterious disease. The original foundation cats for the breeding colony were obtained from different regions in the USA and the breeders did not have cooperative breeding programs. Thus, the Persian PRA may be widespread, although no additional cats have been identified since the establishment of the colony. Likely, with the low activity temperament of the Persian and the strong adaptive ability of blind cats, many owners may not realize that their cats may have vision loss. Therefore, all the greater concern for breeds using Persians as they need to monitor vision loss.

A parametric linkage analysis, assuming a recessive model, showed linkage on chromosome E1 and significant LOD scores (LOD ~14) for a region of 1.75 Mb. The higher LOD scores obtained using parametric linkage analysis reflect the expected increase in power over that of the non-parametric analyses when the mode of inheritance is known.

To exploit the SNP dataset and pedigree structure to the greatest possible extent, three genome-wide association analyses were conducted. The first was a test of linkage in the presence of an association in trios that is referred to as transmission disequilibrium test (TDT) (Spielman et al. 1993). A signal of significant association via TDT strongly indicated chromosome E1. The second test of linkage given association between sib-pairs is referred to as transmission disequilibrium test among sib-pairs (sib-TDT) (Spielman and Ewens 1998). In accordance with the findings of the TDT, sib-TDT suggested the same region on chromosome E1. Finally, the case–control association analysis using all individuals of the pedigree also suggested chromosome E1 in accordance with the previous analyses. However, the genomic inflation suggested possible population substructure within the pedigree. Upon removal of distantly clustered control individuals, the association signal had less stochastic noise and the genomic inflation was reduced significantly.

Systematic and methodical comparison across the three association analyses was not possible because each analysis contained different numbers of individuals. However, all analyses consistently suggested the region on chromosome E1 and with similar pattern of *P*_raw_ values. When comparing the results of the parametric linkage analysis to the association analyses, the significant region was more refined in the latter analyses.

The E1 SNP position reassignment was essential for the current study as many E1 SNPs were previously erroneously assigned to the “unknown” chromosome bin. After reassignment (previous analysis data not shown), the GWAS was more significant and the cat assembly was more consistent with the corresponding human region. The GWAS appears potentially as biphasic, most likely suggesting the genome assembly may still need improvements in the region, or perhaps the disease involves a large inversion. The region of high association and linkage (~1.36 Mb) harbors 22 candidate genes with different degrees of involvement in ocular development and function. This region corresponds to the human region 17p13, which contains mutations responsible for several retinal disorders (Balciuniene et al. 1995; Camuzat et al. 1996; Hameed et al. 2000). Three human genes are markedly associated with retinal degeneration and represent likely candidates. *PITPNM3*, a human homolog of the *Drosophila* *retinal degeneration B* gene (*rdgB*) is associated with autosomal dominant cone dystrophy through a missense mutation (Kohn et al. 2007). Mutations within *AIPL1* (*aryl*-*hydrocarbon interacting protein*-*like 1*) cause Leber congenital amaurosis (Sohocki et al. 2000a, b). Although not found in cats near the genes mentioned earlier, *GUCY2D*, *guanylate cyclase 2D*, maps to same region of *PITPNM3* and *AIPL1* in humans. Mutations within *GUCY2D* are thought to be responsible for autosomal dominant progressive cone degeneration (Kitiratschky et al. 2008). These candidate genes should receive priority in future molecular investigations.

The additional candidate gene is *ARRB2*, *arrestin beta 2*, which is involved in retinal degeneration in *Drosophila* (Alloway et al. 2000). Also, transgenic mice lacking this gene exhibited subnormal rod photo responses (Xu et al. 1997). *ARRB2* has 15 exons in humans. Fourteen exons were directly sequenced in the cat, which did not reveal any candidate mutations of Persian PRA (data not shown). However, exon 1, which in mainly UTR and only 23 bases of coding, has not been analyzed, as well as other regulatory elements of the gene. Thus, *ARRB2* cannot yet be fully excluded as a candidate for Persian PRA. The remaining candidate genes shown in Table S4 are of lower priority and should be investigated if no mutations are identified in the most likely candidates.

This study localized a spontaneously occurring autosomal recessive photoreceptor degeneration of Persian cats to a 1.3 Mb region on cat chromosome E1, which is homologous to human chromosome 17. The identified region is gene-rich and has several candidates for molecular investigation. Regional exome capture, whole genome sequencing and additional recombinants from unrelated cats would support the mutation detection. Each analysis, linkage, case–control, and TDT, localized the trait to the same region, suggesting each had sufficient power using the cats available and the density of the current cat DNA array despite the high genome inflation observed in the case–control analysis. Finally, this study considers the linkage and TDT as the most valid methods for the analysis of this population.

## Electronic supplementary material

Below is the link to the electronic supplementary material.

Supplementary material 1 (DOC 244 kb)

Supplementary material 2 (DOC 1696 kb)

Supplementary material 3 (XLS 1388 kb)
