# Supplementary material for: Genome-wide association and linkage analyses localize a progressive retinal atrophy locus in Persian cats
Source: Mamm Genome. 2014 Apr 29;25(7):354–62. doi: 10.1007/s00335-014-9517-z (PMC4105591; doi:10.1007/s00335-014-9517-z)
Supplement: Supplementary file 1 — Supplementary material 1 (DOC 244 kb) [file 335_2014_9517_MOESM1_ESM.doc]

Table S1: Summary of the remapping of the 63K cat array SNPs to ICGSG Felis catus 6.2/felCat5

| **Chr.** | **Original total No. of SNPs** | **Previously on Chr.** | **No. from Chr. Unk** | **No. from other Chrs.** | **No. removed from Chr.** | **Current total No. of SNPs** |
| --- | --- | --- | --- | --- | --- | --- |
| A1 | 5750 | 5572 | 614 | 158 | 185 | 6344 |
| A2 | 3909 | 3829 | 525 | 73 | 80 | 4427 |
| A3 | 3206 | 3177 | 237 | 30 | 29 | 3444 |
| B1 | 4268 | 4189 | 765 | 129 | 93 | 5083 |
| B2 | 3181 | 3082 | 639 | 97 | 110 | 3818 |
| B3 | 3327 | 3066 | 617 | 71 | 268 | 3754 |
| B4 | 3430 | 3315 | 277 | 165 | 115 | 3757 |
| C1 | 5388 | 5141 | 458 | 173 | 247 | 5772 |
| C2 | 3819 | 3706 | 441 | 74 | 120 | 4221 |
| D1 | 2843 | 2775 | 282 | 61 | 70 | 3118 |
| D2 | 2106 | 2074 | 266 | 35 | 33 | 2375 |
| D3 | 2339 | 2249 | 224 | 85 | 91 | 2558 |
| D4 | 2163 | 2120 | 286 | 79 | 43 | 2485 |
| E1 | 1285 | 1185 | 349 | 24 | 101 | 1558 |
| E2 | 1642 | 1548 | 76 | 52 | 94 | 1676 |
| E3 | 1070 | 997 | 39 | 59 | 67 | 1095 |
| F1 | 1480 | 1441 | 355 | 19 | 39 | 1815 |
| F2 | 1965 | 1941 | 211 | 15 | 29 | 2167 |
| X | 2741 | 2600 | 48 | 88 | 178 | 2736 |
| Unk. | 6985 | 184 | - | 510 | 6709 | 694 |
| **Total** | **62897** | **54191** | **6709** | **1997** | **8701** | **62897** |
| **%** | **100** | **86.16** | **10.67** | **3.18** | **13.83** | **100** |

Table S2: Updated SNP positions for the 63K cat array based on 6.2 cat genome assembly

Table S3: Top 40 SNPs in the parametric genome-wide linkage analysis

| **No.** | **CHR** | **SNP name** | **Position (bp)** | **LOD*** | **ALPHA**** | ***h*-LOD***** |
| --- | --- | --- | --- | --- | --- | --- |
| 1 | E1 | chrUn5.6766609 | 1751066 | 14.1021 | 1 | 14.1021 |
| 2 | E1 | chrUn5.6827940 | 1812918 | 14.1021 | 1 | 14.1021 |
| 3 | E1 | chrUn5.6839723 | 1831172 | 14.1021 | 1 | 14.1021 |
| 4 | E1 | chrUn5.6912692 | 1912858 | 14.1021 | 1 | 14.1021 |
| 5 | E1 | chrUn5.6942249 | 1932982 | 14.1021 | 1 | 14.1021 |
| 6 | E1 | chrUn5.6035236 | 1005256 | 14.102 | 1 | 14.102 |
| 7 | E1 | chrUn5.6096257 | 1067642 | 14.102 | 1 | 14.102 |
| 8 | E1 | chrUn5.6133983 | 1106562 | 14.102 | 1 | 14.102 |
| 9 | E1 | chrUn5.6185290 | 1154788 | 14.102 | 1 | 14.102 |
| 10 | E1 | chrUn5.6262913 | 1232384 | 14.102 | 1 | 14.102 |
| 11 | E1 | chrUn5.6307120 | 1275288 | 14.102 | 1 | 14.102 |
| 12 | E1 | chrUn5.6334444 | 1288658 | 14.102 | 1 | 14.102 |
| 13 | E1 | chrUn5.6346473 | 1315110 | 14.102 | 1 | 14.102 |
| 14 | E1 | chrUn5.6410839 | 1379688 | 14.102 | 1 | 14.102 |
| 15 | E1 | chrUn5.6467522 | 1447452 | 14.102 | 1 | 14.102 |
| 16 | E1 | chrUn5.6481762 | 1452354 | 14.102 | 1 | 14.102 |
| 17 | E1 | chrUn5.6503134 | 1476010 | 14.102 | 1 | 14.102 |
| 18 | E1 | chrUn5.6605276 | 1576124 | 14.102 | 1 | 14.102 |
| 19 | E1 | chrUn5.6654818 | 1628586 | 14.102 | 1 | 14.102 |
| 20 | E1 | chrUn5.6713247 | 1695928 | 14.102 | 1 | 14.102 |
| 21 | E1 | chrUn5.5553521 | 517030 | 14.1019 | 1 | 14.1019 |
| 22 | E1 | chrUn5.5612362 | 578796 | 14.1019 | 1 | 14.1019 |
| 23 | E1 | chrUn5.5683154 | 647314 | 14.1019 | 1 | 14.1019 |
| 24 | E1 | chrUn5.5751502 | 713552 | 14.1019 | 1 | 14.1019 |
| 25 | E1 | chrUn5.5779779 | 742232 | 14.1019 | 1 | 14.1019 |
| 26 | E1 | chrUn5.5819438 | 781220 | 14.1019 | 1 | 14.1019 |
| 27 | E1 | chrUn5.5846986 | 808916 | 14.1019 | 1 | 14.1019 |
| 28 | E1 | chrUn5.5865430 | 829890 | 14.1019 | 1 | 14.1019 |
| 29 | E1 | chrUn5.5486460 | 451267 | 14.1005 | 1 | 14.1005 |
| 30 | E1 | chrUn5.5447450 | 413622 | 14.0997 | 1 | 14.0997 |
| 31 | E1 | chrUn5.5399253 | 364520 | 14.0986 | 1 | 14.0986 |
| 32 | E1 | chrUn5.5280164 | 246372 | 14.096 | 1 | 14.096 |
| 33 | E1 | chrD1.18701 | 227952 | 14.0956 | 1 | 14.0956 |
| 34 | E1 | chrD1.27736 | 221782 | 14.0955 | 1 | 14.0955 |
| 35 | E1 | chrD1.111564 | 179844 | 14.085 | 1 | 14.085 |
| 36 | E1 | chrUn5.7087155 | 2076816 | 13.1739 | 1 | 13.1739 |
| 37 | E1 | chrUn5.7231276 | 2217474 | 5.1386 | 0.8052 | 8.5946 |
| 38 | E1 | chrUn5.7293975 | 2293660 | 3.391 | 0.7147 | 7.7072 |
| 39 | E1 | chrUn5.7489650 | 2495632 | 5.4613 | 0.7265 | 7.6392 |
| 40 | E1 | chrUn5.7948667 | 2997440 | 2.6599 | 0.6986 | 7.5127 |

* Multi-point LOD scores

** An estimate of the proportion of linked families

*** Maximum Heterogeneity LOD score

Table S4: List of the human coding genes in the Persian PRA identified region.

| **#** | **Gene name** | **Symbol** | **start** | **end** | **Ref** |
| --- | --- | --- | --- | --- | --- |
| 1 | Mediator complex subunit 31 | MED31 | 716470 | 724348 | - |
| 2 | Thioredoxin domain containing 17 | TXNDC17 | 723385 | 726710 | - |
| 3 | KIAA0753 | KIAA0753* | 736545 | 765228 | (Au*do, et a*l., 2012) |
| 4 | PITPNM family member 3 | PITPNM3* ^1^ | 795681 | 851951 | (Ko*hn, et a*l., 2007) |
| 5 | Family with sequence similarity 64, member A | FAM64A* | 852309 | 856348 |  |
| 6 | Aryl hydrocarbon receptor interacting protein-like 1 | AIPL1* ^2^ | 861218 | 868260 | (Sohoc*ki, et a*l., 2000) |
| 7 | WSC domain containing 1 | WSCD1 | 1011130 | 1033440 |  |
| 8 | MIND kinetochore complex component, | MIS12 | 1311503 | 1313010 |  |
| 9 | Derlin 2 | DERL2 | 1317211 | 1327587 |  |
| 10 | DEAH (Asp-Glu-Ala-His) box polypeptide 33 | DHX33 | 1335484 | 1349146 |  |
| 11 | Zinc finger protein 33A | ZNF33A | 1345075 | 1563789 |  |
| 12 | Complement component 1, q subcomponent binding protein | C1QBP* | 1352357 | 1356115 | (Lichans*ka, et a*l., 2001) |
| 13 | RPA interacting protein | RPAIN | 1356114 | 1363727 |  |
| 14 | Nucleoporin 88kDa | NUP88* | 1363653 | 1388890 | (Davulu*ri, et a*l., 2008; Lichans*ka, et a*l., 2001) |
| 15 | Rabaptin, RAB GTPase binding effector protein 1 | RABEP1* | 1389107 | 1492003 | (Ishikawa and Marshall, 2011; Kubo*ta, et a*l., 2002; Sta*rr, et a*l., 2004) |
| 16 | SLP adaptor and CSK interacting membrane protein | SCIMP | 1517741 | 1436087 |  |
| 17 | ZFP3 zinc finger protein | ZFP3 | 1562809 | 1564089 |  |
| 18 | Inhibitor of CDK, cyclin A1 interacting protein 1 | INCA1 | 1614229 | 1615935 |  |
| 19 | Calmodulin binding transcription activator 2 | CAMTA2* | 1617798 | 1631261 | (Finkl*er, et a*l., 2007) |
| 20 | Sperm associated antigen 7 | SPAG7 | 1631401 | 1635302 |  |
| 21 | Enolase 3 (beta, muscle) | ENO3* | 1635843 | 1640667 | (Leu*ng, et a*l., 2007) |
| 22 | Profilin 1 | PFN1 | 1641534 | 1642704 |  |
| 23 | Ring finger protein 167 | RNF167* | 1643211 | 1647452 | (Yama*da, et a*l., 2013) |
| 24 | Glycoprotein Ib (platelet), alpha polypeptide | GP1BA* | 1653057 | 1655193 | (Moham*ed, et a*l., 2009) |
| 25 | Cholinergic receptor, nicotinic, epsilon (muscle) | CHRNE* | 1673489 | 1675201 | (D*as, et a*l., 2005) |
| 26 | Misshapen-like kinase 1 | MINK1* | 1682211 | 1696951 | (Baye and Link, 2008) |
| 27 | Phospholipase D2 | PLD2* | 1730503 | 1742048 | (L*ee, et a*l., 2001) |
| 28 | Proteasome (prosome, macropain) subunit, beta type, 6 | PSMB6* | 1751042 | 1753071 | (Die*hn, et a*l., 2005) |
| 29 | Glycolipid transfer protein domain containing 2 | GLTPD2 | 1758633 | 1759881 |  |
| 30 | Vitelline membrane outer layer 1 homolog (chicken) | VMO1 | 1761815 | 1762800 |  |
| 31 | Transmembrane 4 L six family member 5 | TM4SF5* | 1764343 | 1770128 | (Moral*es, et a*l., 2013) |
| 32 | Zinc finger, MYND-type containing 15 | ZMYND15 | 1787379 | 1791810 |  |
| 33 | Chemokine (C-X-C motif) ligand 16 | CXCL16* | 1793455 | 1795250 | (Hijio*ka, et a*l., 2008; Roes*ch, et a*l., 2008) |
| 34 | Mediator complex subunit 11 | MED11 | 1796545 | 1797876 |  |
| 35 | Arrestin, beta 2 | ARRB2* | 1806085 | 1811621 | (Larhamm*ar, et a*l., 2009) |
| 36 | Proline, glutamate and leucine rich protein 1 | PELP1* | 1830437 | 1843347 | (R*en, et a*l., 2012) |
| 37 | Arachidonate 15-lipoxygenase | ALOX15* | 1857626 | 1948580 | (Gronert, 2005; Q*in, et a*l., 2008) |
| 38 | Ribonuclease, RNase K | RNASEK | 1951150 | 1952669 |  |
| 39 | B-cell CLL/lymphoma 6, member B | BCL6B | 1960986 | 1966513 |  |
| 40 | C-type lectin domain family 10, member A | CLEC10A | 1994343 | 1996875 |  |
| 41 | Asialoglycoprotein receptor 1 | ASGR1* | 1994405 | 2052425 | (Ch*en, et a*l., 2012) |
| 42 | Discs, large homolog 4 (Drosophila) | DLG4* | 2062133 | 2083657 | (Ludford-Menti*ng, et a*l., 2002; Mclaughl*in, et a*l., 2003) |

*Candidate genes involved in the retina

^1^ A missense mutation (Q626H) causes an autosomal dominant eye disease affecting the cone

^2^ Mutations (W278X, C239R, Del 1053-1064) cause autosomal dominant and recessive diseases affecting the cone or rod.

Reference:

Audo I, Bujakowska K, Orhan E, Poloschek CM, Defoort-Dhellemmes S, Drumare I, Kohl S, Luu TD, Lecompte O, Zrenner E*, et al.* 2012 Whole-exome sequencing identifies mutations in GPR179 leading to autosomal-recessive complete congenital stationary night blindness. Am J Hum Genet. 90(2):321-330.

Baye LM, Link BA. 2008 Nuclear migration during retinal development. Brain Res. 1192:29-36.

Chen YH, Chen JY, Chen YW, Lin ST, Chan HL. 2012 High glucose-induced proteome alterations in retinal pigmented epithelium cells and its possible relevance to diabetic retinopathy. Mol Biosyst. 8(12):3107-3124.

Das AV, Edakkot S, Thoreson WB, James J, Bhattacharya S, Ahmad I. 2005 Membrane properties of retinal stem cells/progenitors. Prog Retin Eye Res. 24(6):663-681.

Davuluri G, Gong W, Yusuff S, Lorent K, Muthumani M, Dolan AC, Pack M. 2008 Mutation of the zebrafish nucleoporin elys sensitizes tissue progenitors to replication stress. PLoS Genet. 4(10):e1000240.

Diehn JJ, Diehn M, Marmor MF, Brown PO. 2005 Differential gene expression in anatomical compartments of the human eye. Genome Biol. 6(9):R74.

Finkler A, Ashery-Padan R, Fromm H. 2007 CAMTAs: calmodulin-binding transcription activators from plants to human. FEBS Lett. 581(21):3893-3898.

Gronert K. 2005 Lipoxins in the eye and their role in wound healing. Prostaglandins Leukot Essent Fatty Acids. 73(3-4):221-229.

Hijioka K, Sonoda KH, Tsutsumi-Miyahara C, Fujimoto T, Oshima Y, Taniguchi M, Ishibashi T. 2008 Investigation of the role of CD1d-restricted invariant NKT cells in experimental choroidal neovascularization. Biochem Biophys Res Commun. 374(1):38-43.

Ishikawa H, Marshall WF. 2011 Ciliogenesis: building the cell's antenna. Nat Rev Mol Cell Biol. 12(4):222-234.

Kohn L, Kadzhaev K, Burstedt MS, Haraldsson S, Hallberg B, Sandgren O, Golovleva I. 2007 Mutation in the PYK2-binding domain of PITPNM3 causes autosomal dominant cone dystrophy (CORD5) in two Swedish families. Eur J Hum Genet. 15(6):664-671.

Kubota S, Kobayashi A, Mori N, Higashide T, McLaren MJ, Inana G. 2002 Changes in retinal synaptic proteins in the transgenic model expressing a mutant HRG4 (UNC119). Invest Ophthalmol Vis Sci. 43(2):308-313.

Larhammar D, Nordstrom K, Larsson TA. 2009 Evolution of vertebrate rod and cone phototransduction genes. Philos Trans R Soc Lond B Biol Sci. 364(1531):2867-2880.

Lee EJ, Min DS, Kang WS, Lee MY, Oh SJ, Chun MH. 2001 The expression and cellular localization of phospholipase D1 in the rodent retina. Brain Res. 905(1-2):240-244.

Leung YF, Ma P, Dowling JE. 2007 Gene expression profiling of zebrafish embryonic retinal pigment epithelium in vivo. Invest Ophthalmol Vis Sci. 48(2):881-890.

Lichanska AM, McGibbon D, Silvestri G, Hughes AE. 2001 A physical and expression map of the D17S1810-D17S1353 region spanning the central areolar choroidal dystrophy locus. Cytogenet Cell Genet. 93(1-2):43-47.

Ludford-Menting MJ, Thomas SJ, Crimeen B, Harris LJ, Loveland BE, Bills M, Ellis S, Russell SM. 2002 A functional interaction between CD46 and DLG4: a role for DLG4 in epithelial polarization. J Biol Chem. 277(6):4477-4484.

McLaughlin BJ, Fan W, Zheng JJ, Cai H, Del Priore LV, Bora NS, Kaplan HJ. 2003 Novel role for a complement regulatory protein (CD46) in retinal pigment epithelial adhesion. Invest Ophthalmol Vis Sci. 44(8):3669-3674.

Mohamed S, Schaa K, Cooper ME, Ahrens E, Alvarado A, Colaizy T, Marazita ML, Murray JC, Dagle JM. 2009 Genetic contributions to the development of retinopathy of prematurity. Pediatr Res. 65(2):193-197.

Morales SA, Telander DG, Leon D, Forward K, Braun J, Wadehra M, Gordon LK. 2013 Epithelial membrane protein 2 (EMP2) controls VEGF expression in ARPE-19 cells. Invest Ophthalmol Vis Sci. **28;54(3):2367-72**.

Qin Q, Patil KA, Gronert K, Sharma SC. 2008 Neuroprotectin D1 inhibits retinal ganglion cell death following axotomy. Prostaglandins Leukot Essent Fatty Acids. 79(6):201-207.

**Ren P, Gong M-z, Wang Z-y, Zhang P, Chen P, Ma W-l, Zhou C-j. 2012 DACH1 Expression in Osteosarcoma and Its Relationship with Proliferation and Angiogenesis. Indian J Surgery.1-6. DOI 10.1007/s12262-01200761-8**

Roesch K, Jadhav AP, Trimarchi JM, Stadler MB, Roska B, Sun BB, Cepko CL. 2008 The transcriptome of retinal Muller glial cells. J Comp Neurol. 509(2):225-238.

Sohocki MM, Perrault I, Leroy BP, Payne AM, Dharmaraj S, Bhattacharya SS, Kaplan J, Maumenee IH, Koenekoop R, Meire FM*, et al.* 2000 Prevalence of AIPL1 mutations in inherited retinal degenerative disease. Mol Genet Metab. 70(2):142-150.

Starr CJ, Kappler JA, Chan DK, Kollmar R, Hudspeth AJ. 2004 Mutation of the zebrafish choroideremia gene encoding Rab escort protein 1 devastates hair cells. Proc Natl Acad Sci U S A. 101(8):2572-2577.

Yamada T, Yang Y, Bonni A. 2013 Spatial organization of ubiquitin ligase pathways orchestrates neuronal connectivity. Trends Neurosci.**36(4):218-26. Epub 2013 Jan 17.**
