# Supplementary material for: Genome-wide association and linkage analyses localize a progressive retinal atrophy locus in Persian cats
Source: Mamm Genome. 2014 Apr 29;25(7):354–62. doi: 10.1007/s00335-014-9517-z (PMC4105591; doi:10.1007/s00335-014-9517-z)
Supplement: Supplementary file 2 — Supplementary material 2 (DOC 1696 kb) [file 335_2014_9517_MOESM2_ESM.doc]

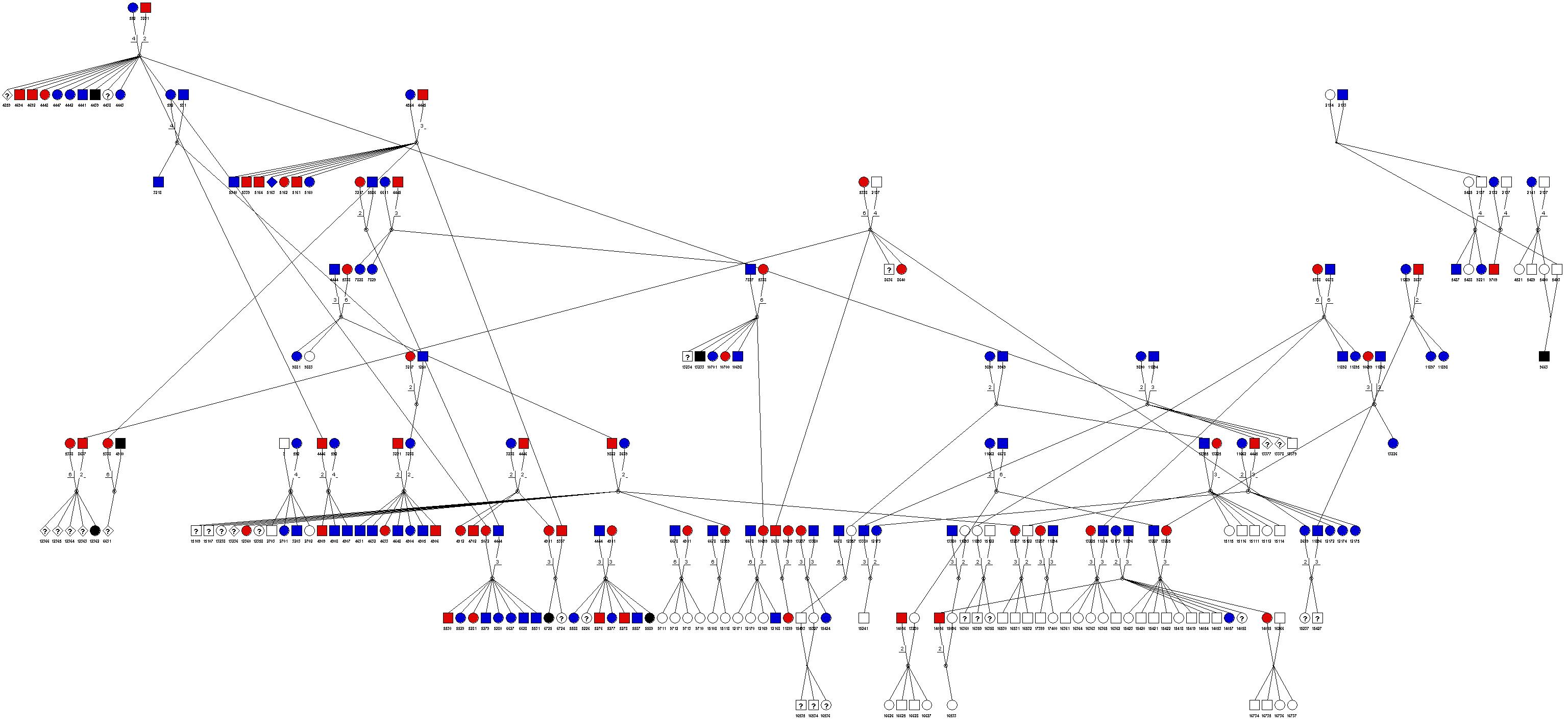


Figure S1 Pedigree of Persian progressive retinal atrophy. The pedigree is composed of 202 cats where unaffected and affected cats are marked by non-filled and black filled symbols, respectively. Unaffected and affected cats included in the study are marked by blue and red filled symbols, respectively. Multiple matings are shown separately for clarity, some individuals are presented more than once.


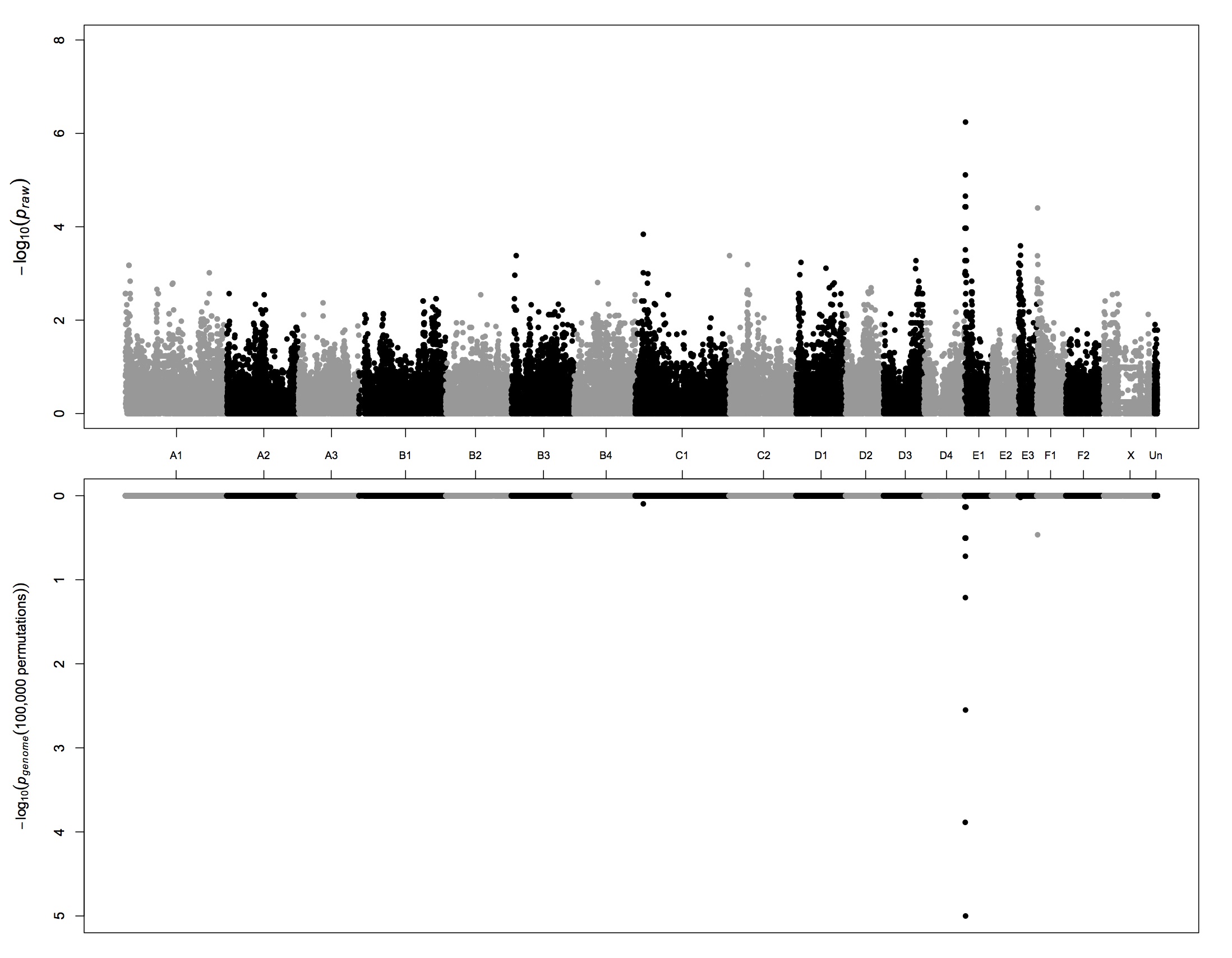


Figure S2 Genome-wide TDT analysis of the Persian PRA. Upper plot represents the *P*_raw_ values of the analysis whereas the lower plot represents the genome-wide significant *P*_genome_ values after 100,000 permutations. Significant association is localized to cat chromosome E1.


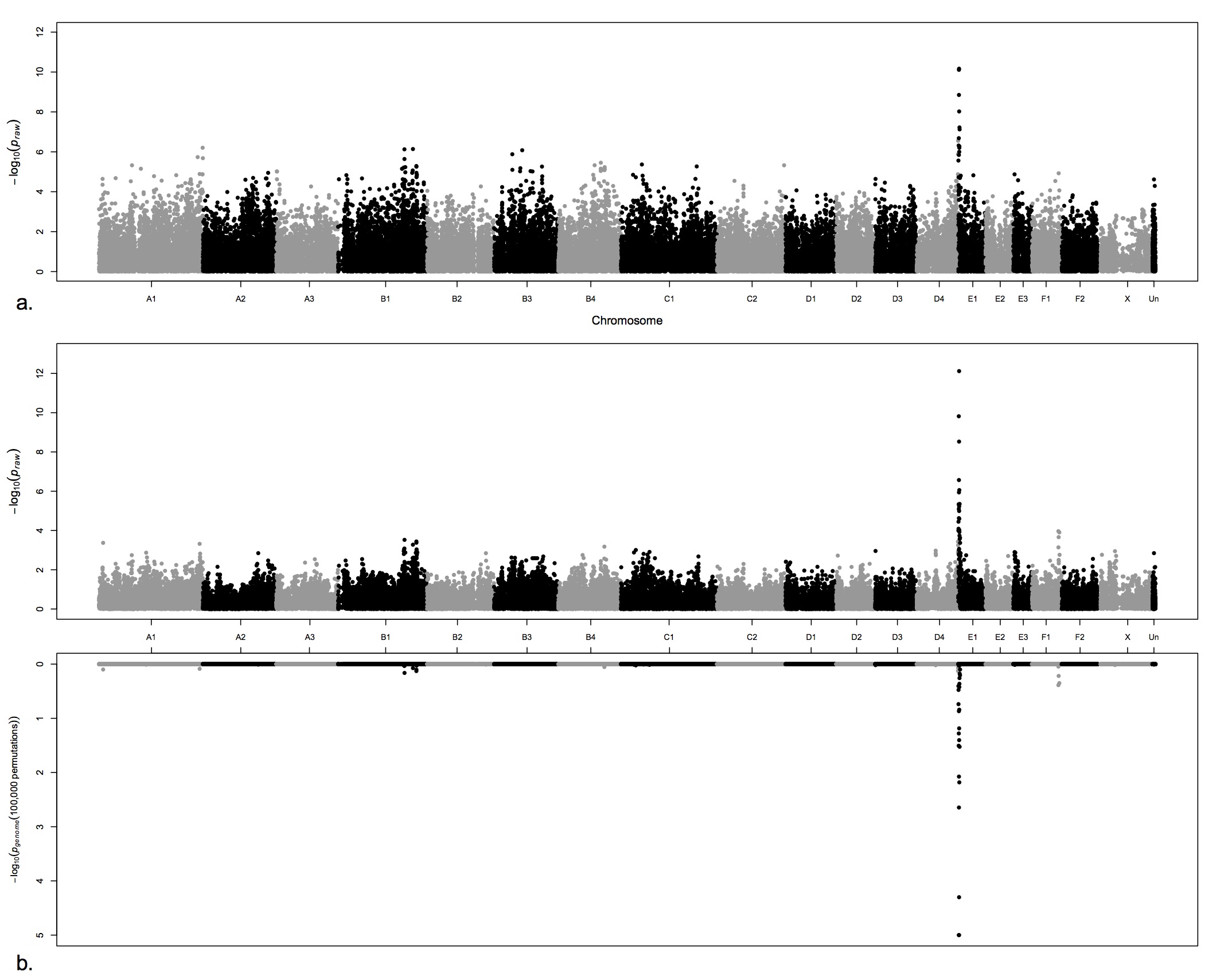


Figure S3 Genome-wide association analysis of the Persian PRA. a) Case-control association analysis using all cats (Figure S7a). b) Case-control analysis using closely clustered cases and controls in MDS (Figure S7b). Upper plot represents the *P*_raw_ values of the analysis whereas the lower plot represents the genome-wide significant *P*_genome_ values after 100,000 permutations.


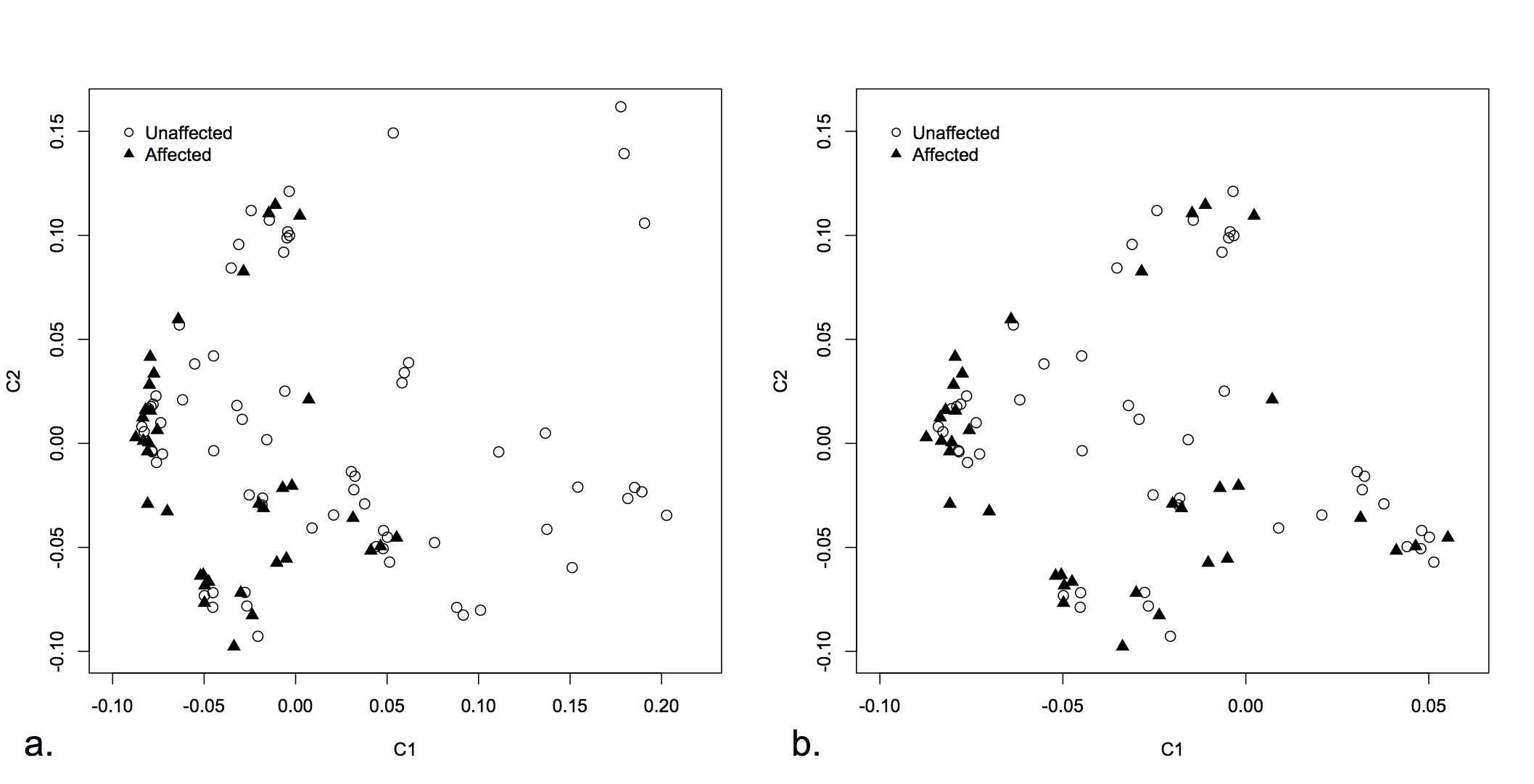


Figure S4 Multi-dimensional scaling (MDS) of the Persian PRA pedigree cats. a) MDS of all individuals in the pedigree (n = 106). b) MDS of selected affected and unaffected cats (n = 106) that cluster more closely after removing distant unaffected cats (n = 20).


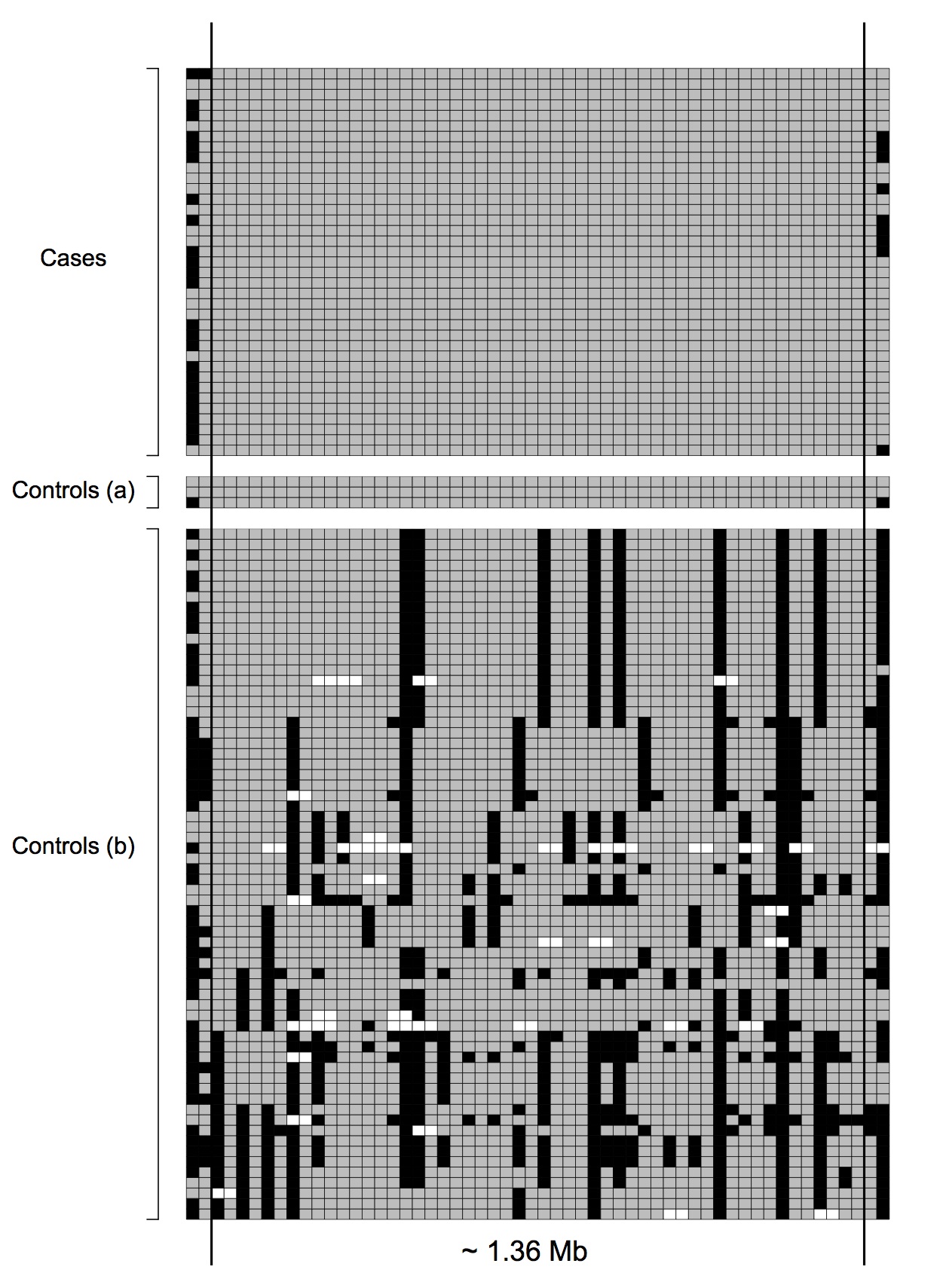


Figure S5 SNP genotypes around the most associated markers on E1. The area spans 28 markers, chrUn5.5683154 (position 647314) to chrUn5.7160649 (position 2164282). Two vertical lines represent a region of single haplotype across all cases that spans 26 markers, chrUn5.5751502 (position 713552) to chrUn5.7087155 (position 2076816). Each SNP genotype is presented by two squares where markers are on the x-axis and individuals on the y-axis. Gray boxes represent the major allele in the cases and black squares represent the minor. White boxes represent lack of genotype data. Controls (a) are three individuals that share the single haplotype of the cases whereas controls (b) do not.
